# Recombinant High-Mobility Group Box 1 (rHMGB1) Promotes NRF2-Independent Mitochondrial Fusion through CXCR4/PSMB5-Mediated Drp1 Degradation in Endothelial Cells

**DOI:** 10.1155/2021/9993240

**Published:** 2021-08-02

**Authors:** Shunrong Zhang, Fei Feng, Jingting Dai, Jia Li, Xiangye Bu, Xiaojie Xie

**Affiliations:** ^1^Department of Cardiology, Second Affiliated Hospital, Zhejiang University School of Medicine, Hangzhou, Zhejiang, China; ^2^Department of Geriatrics, Affiliated Hangzhou First People's Hospital, Zhejiang University School of Medicine, Hangzhou, Zhejiang, China

## Abstract

Mitochondrial dynamics plays an important role in maintaining normal endothelial cell function and in the pathogenesis of cardiovascular disease. It is not identified whether high-mobility group box 1 (HMGB1), a representative damage-associated molecular pattern (DAMP) molecule, could influence mitochondrial dynamics in endothelial cells. The objective of this study is to clarify the effect of HMGB1 on mitochondrial dynamics in endothelial cells and the underlying mechanism. EA.hy926 human endothelial cells were incubated with recombinant HMGB1 (rHMGB1); mitochondrial morphology was observed with a confocal microscope and transmission electron microscope (TEM). The expression of dynamin-related protein 1 (Drp1), Mitofusin 1 (Mfn1), Mitofusin 2 (Mfn2), Optic atrophy 1 (Opa1), phosphatase and tensin homolog- (PTEN-) induced kinase 1 (PINK1), NOD-like receptor 3 (NLRP3), caspase 1, cleaved caspase 1, 20S proteasome subunit beta 5 (PSMB5), and antioxidative master nuclear factor E2-related factor 2 (NRF2) and the concentration of interleukin 1*β* (IL-1*β*) were determined. Specific inhibitors C29, TAK-242, FPS-ZM1, AMD3100, and epoxomicin were used to block toll-like receptor 2 (TLR2), toll-like receptor 4 (TLR4), receptor for advanced glycation end products (RAGE), C-X-C-chemokine receptor 4 (CXCR4), and PSMB5, respectively. siRNAs were used to silence the expression of NRF2. rHMGB1 promoted mitochondrial fusion in endothelial cells, while no significant proinflammatory effects were found. The expression of mitochondrial fission protein Drp1 and phosphorylated subtypes p-Drp1-S616 and p-Drp1-S637 were all downregulated; no significant expression changes of PINK1 and Mfn1, Mfn2, and Opa1 were found. Inhibition of CXCR4 but not TLR4, RAGE, or TLR2 reversed rHMGB1-induced Drp1 downregulation and mitochondrial fusion. Interestingly, inhibition of TLR4 with TAK-242 promoted Drp1 downregulation and mitochondrial fusion. rHMGB1 increased the expression of NRF2 and PSMB5; inhibition of PSMB5 but not silencing NRF2 abolished rHMGB1-induced Drp1 downregulation and mitochondrial fusion. These results indicate that rHMGB1 promotes NRF2 independent mitochondrial fusion via CXCR4/PSMB5 pathway-mediated Drp1 proteolysis. rHMGB1 may influence mitochondrial and endothelial function through this effect on mitochondrial dynamics.

## 1. Introduction

Vascular endothelium, building the inner layer of capillaries and blood vessels, is the largest organ in the body [1]. As a highly active metabolic and endocrine organ [2], the endothelium can produce a variety of different bioactive molecules and plays a crucial role in the regulation of hemostasis, blood flow, maintenance of vascular architecture, control of thrombosis and thrombolysis, mediation of platelet and leukocyte interaction with the vessel wall, and the regulation of vascular tone and growth of blood vessels [3, 4].

Endothelial dysfunction plays an important role in the pathogenesis of many cardiovascular diseases(CVDs), including atherosclerosis [5], hypertension [6], pulmonary hypertension [7], stroke [8], heart failure [9], and diabetic vascular complications [10]. In fact, one of the earliest detectable changes in the development of atherosclerosis is endothelial cell activation and dysfunction at lesion-prone areas of the arterial vasculature [11]. Endothelial dysfunction is characterized by imbalanced vasodilation and vasoconstriction, elevated reactive oxygen species (ROS), deficiency of nitric oxide (NO) bioavailability, disruption of endothelial barrier permeability [12], and a transformation to proinflammatory phenotype. Proinflammatorily activated endothelium secretes a variety of chemokines, such as intercellular cell adhesion molecule-1 (ICAM-1), vascular cell adhesion molecule-1 (VCAM-1), interleukin 1*β* (IL-1*β*), interleukin 8 (IL-8), monocyte chemotactic protein 1 (MCP-1), and granulocyte-monocyte stimulating factor (GM-CSF), promoting monocyte/macrophage transendothelial migration, proliferation of vascular smooth muscle cells (VSMCs), atherosclerotic lesion formation, progression, and rupture [13].

High-mobility group box 1 (HMGB1), also known as high-mobility group protein 1 (HMG-1) and amphoterin, is a highly conserved nonhistone nuclear protein involved in transcription regulation, DNA replication and repair, and nucleosome assembly [14] and is passively released by necrotic tissues or actively secreted by stressed cells. Extracellular HMGB1 acts as a typical damage-associated molecular pattern (DAMP) molecule or alarmin, to promote a variety of cellular responses including inflammation by binding to different receptors, such as toll-like receptors 2 and 4 (TLR2 and TLR4), receptor for advanced glycation end products (RAGE), and C-X-C-chemokine receptor 4 (CXCR4) [15, 16]. Though a research hotspot, the role of HMGB1 in CVDs is very intriguing; both harmful and beneficial effects were reported [14]. As a distinct proinflammatory cytokine, HMGB1 contributed to the pathogenesis of myocardial ischemia-reperfusion injury [17], heart failure [18], and diabetes [19]; however, a series of beneficial effects of HMGB1 in CVDs were also found, such as boosting myocardial regeneration and repair after infarction [20, 21] and protecting against ischemia-reperfusion injury [22]. As for the endothelium, the contradictory effects of HMGB1 were also found; some reported that HMGB1 induced endothelial dysfunction and inflammation [23, 24], inhibited endothelial cell migration [25], and enhanced LDL transcytosis in endothelial cells to promote the pathogenesis of atherosclerosis [26], while others reported that HMGB1 promoted angiogenesis in endothelial cells [27]. Recently, Zhou et al. [28] have reported that endothelial-specific deletion of HMGB1 in mice increased reactive oxygen species (ROS) production and blood pressure and retarded endothelium-dependent relaxation (EDR) and ischemia recovery, demonstrating the crucial role of HMGB1 in maintaining healthy endothelial function.

Mitochondrion is not a static organelle but can dynamically reconstruct its shape by continuous fusion and fission to adapt to the change of homeostasis of cells; this dynamic changing process is called mitochondrial dynamics [29]. Balanced mitochondrial fission-fusion dynamics plays an essential role in mitochondrial quality control, cellular metabolism, homeostasis, and stress responses [30]. Mitochondrial dynamics is delicately orchestrated by several fission and fusion mediators; the former includes Mitofusin 1 (Mfn1), Mitofusin 2 (Mfn2), and Optic atrophy 1 (Opa1) [31]; the latter mainly consists of dynamin-related protein 1 (Drp1) and its several adaptors, such as mitochondrial fission 1 protein (Fis1) and mitochondrial dynamics proteins of 49 and 51 kDa (MiD49 and MiD51) [32]. Additionally, the phosphatase and tensin homolog- (PTEN-) induced kinase 1 (PINK1), though primarily a modulator of mitophagy, also plays an important role in regulating mitochondrial dynamics [33]. Disruption of mitochondrial dynamics is associated with a range of human diseases including atherosclerotic cardiovascular disease (ASCVD) [34], heart failure [35], myocardial ischemia-reperfusion (IR) injury [36], and inflammatory diseases [37]. Studies have reported that altered mitochondrial dynamics resulted in endothelial dysfunction [38, 39].

However, whether the aforementioned typical DAMP molecule HMGB1 can influence mitochondrial dynamics in endothelial cells is not clear. In light of the important role of mitochondrial dynamics in maintaining normal mitochondrial and cellular function and in the pathogenesis of CVDs and inflammatory diseases, it is worthful to clarify the impact of HMGB1 on mitochondrial dynamics in endothelial cells. In this study, we detected the influence of recombinant HMGB1 on mitochondrial dynamics and the underlying mechanism in EA.hy926 endothelial cells.

## 2. Materials and Methods

### 2.1. Cell Culture

Human endothelial EA.hy926 cells (Cat# 3131C0001000200039; Shanghai cell bank of Chinese Academy of Sciences) were cultured in high-glucose DMEM (REF11965-092, Gibco) with 10% FBS (REF10099-141, Gibco) at 37°C under a humidified 95% air and 5% CO_2_ atmosphere. EA.hy926 endothelial cells were transferred and seeded in 6-well plates at a density of 1.5 × 10^5^/well; when cells grew to 80% confluence, human recombinant HMGB1 (rHMGB1, Cat# H4652; Sigma) with different final concentrations (0 *μ*g/ml, 0.1 *μ*g/ml, 0.5 *μ*g/ml, and 1 *μ*g/ml) was added to incubate for 24 hours. For mechanism exploration, cells were preconditioned with specific antagonists for 1 hour prior to incubation with rHMGB1 (1 *μ*g/ml) for 24 hours, respectively. The preconditioned agents were listed as TLR4-specific antagonist TAK-242 (1 *μ*M, Cat# HY-11109; MCE), TLR2-specific antagonist C29 (10 *μ*M, Cat# HY-100461; MCE), RAGE-specific antagonist FPS-ZM1 (1 *μ*M, Cat# HY-19370; MCE), CXCR4-specific antagonist AMD3100 (5 *μ*M, Cat# HY-50912; MCE), and proteasome-selective inhibitor epoxomicin (10 *μ*M, Cat# HY-13821; MCE).

### 2.2. Cell Transfection

Cells were seeded into 6-well plates (1 × 10^5^ cells/well) to ensure 30-50% confluence in the next day and then transfected with NRF2 siRNAs or vehicles as negative control, respectively. The sequences of NRF2 siRNA duplexes and vehicles were listed in Table S1. Cells were transfected with a matching siRNA-Mate (Cat# G04002; Gene Pharma) for 48 hours to determine the efficiency by western blot before the following experiments. All the data were quantified independently by two observers that were blinded to the study design.

### 2.3. Western Blot

Cell lysate samples were prepared from cells in RIPA solution (Cat# FD009; Fude) supplemented with protease inhibitor (Cat# FD1001; Fude) and protein phosphatase inhibitor (Cat# FD1002; Fude). Denatured cell lysates were resolved by SDS-PAGE and transferred onto a polyvinylidene fluoride (PVDF) filter membrane. After transfer, membranes were blocked in 5% (wt/vol) nonfat dry milk diluted in TBST. Membranes were incubated with primary antibodies against Mfn1 (1 : 1000, Cat# 14739; CST), Mfn2 (1 : 1000, Cat# 11925; CST), Drp1 (1 : 1000, Cat# 5391; CST), p-Drp1-S616 (1 : 500, Cat# 3455; CST), p-Drp1-S637 (1 : 500, Cat# 4867; CST), Opa1 (1 : 1000, Cat# ab157457; Abcam), PINK1 (1 : 1000, Cat# 6946; CST), NRF2 (1 : 1000, Cat# ab62352; Abcam), NLRP3 (1 : 1000, Cat# ab210491; Abcam), caspase 1 (1 : 1000, Cat# ab207802; Abcam), cleaved caspase 1 (P20) (1 : 1000, Cat# 4199; CST), PSMB5 (1 : 1000, Cat# abs115883; Absin), GAPDH (1 : 3000, Cat# FD0063; Fude), *β*-actin (1 : 1000, Cat# FD0060; Fude), and *β*-Tubulin (1 : 1000, Cat# A01030; Abbkine) overnight at 4°C and subsequently incubated with horseradish peroxidase- (HRP-) conjugated secondary antibodies which were detected by enhanced chemiluminescence (Cat# FD802; Fude). Immunoblots were analyzed using ImageJ 5.0 software (NIH; MD).

### 2.4. Immunofluorescent Staining

Cells seeded on 12-well plates were fixed with 4% paraformaldehyde (30 minutes), permeabilized with 0.5% Triton X-100 for 5 minutes, and then blocked with 1% BSA in 0.1% PBS-Tween 20 for 1 hour. The cells were then incubated overnight at 4°C with primary antibodies against CD31 (1 : 300, Cat# ab9498, Abcam) or vWF (1 : 500, Cat# ab154193, Abcam), followed by incubation with secondary antibody Donkey anti-Mouse IgG-Alexa Fluor 488 (1 : 1000, Cat# abs20014, Absin) for CD31 or Donkey anti-Rabbit IgG-Alexa Fluor 594 (1 : 1000, Cat# abs20021, Absin) for vWF. Nuclear DNA was labelled with Hoechst (4 *μ*g/ml, Cat# BL801A, Biosharp). Images were investigated under an inverted fluorescence microscope (Ix71, Olympus; Tokyo).

### 2.5. Quantitative RT-PCR Analysis

Total RNA was reversely transcribed with a cDNA Synthesis kit (Cat# RR037A; Takara Bio), and quantitative PCR (qPCR) was performed to quantify mRNA abundance using a SYBR Green PCR Premix (Cat# RR420A; Takara Bio) on an Applied Biosystem cycler. Data were analyzed using the *ΔΔ*Ct method and GAPDH as internal control. Primers used in this study were listed in Table S2.

### 2.6. Enzyme-Linked Immunosorbent Assay (ELISA)

Interleukin 1*β* (IL-1*β*) concentrations of the supernatant in cell culture were measured with ELISA kits according to the manufacturer's recommendation (Cat# DLB50; R&D).

### 2.7. Transmission Electron Microscopy (TEM)

Cells were fixed in 2.5% glutaraldehyde and then postfixed in 1% osmium tetroxide, dehydrated in ethanol of gradient concentrations, and embedded in Sparr resin for electron microscopy. Sections were double-stained with uranyl acetate and alkaline lead citrate and then examined with a transmission electron microscope (TECNAI 10, Philips; Amsterdam). Ten cells of sections in every group (magnification ×5900) were randomly included to count the mitochondrial numbers of cells and get the average. For comparison of average mitochondrial areas, at least 30 mitochondria of 10 cells for every group were calculated and then analyzed using ImageJ 5.0 software (NIH; MD).

### 2.8. Fluorescence Tracing

Cells plated on glass-bottomed dishes (35 mm) were incubated with MitoTracker Green FM (20 *μ*M, Cat# 40742ES50; Yeasen Biotech) in DMEM with 10% FBS for 20 minutes at 37°C. Fluorescence stained cells were analyzed using confocal laser microscopy with a 63x objective (SP8, Leica; Wetzlar).

### 2.9. Statistical Analysis

Data were shown as the mean ± standard deviation (SD). SPSS version 17.0 was used for statistical analyses. To compare continuous response variables between two groups, unpaired two-tailed Student's *t*-test was used for normally distributed variables that passed the equal variance test, and a Mann-Whitney *U* test was performed for variables not passing either normality or equal variance test. *P* < 0.05 was considered statistically significant.

## 3. Results

### 3.1. Exogenous rHMGB1 Incubation Altered Mitochondrial Morphology in EA.hy926 Cells

EA.hy926 cells are a human vascular endothelial cell line presenting typical characteristics of human primary endothelial cells. To characterize EA.hy926 endothelial cells, we first detected endothelial-specific markers CD31 (cluster of differentiation 31) and vWF (von Willebrand factor) with immunofluorescent staining. As expected, both CD31 and vWF were strongly positive in EA.hy926 cells (Figures S1(a) and S1(b)), demonstrating the endothelial characteristics of EA.hy926 cells.

**Figure 1:**
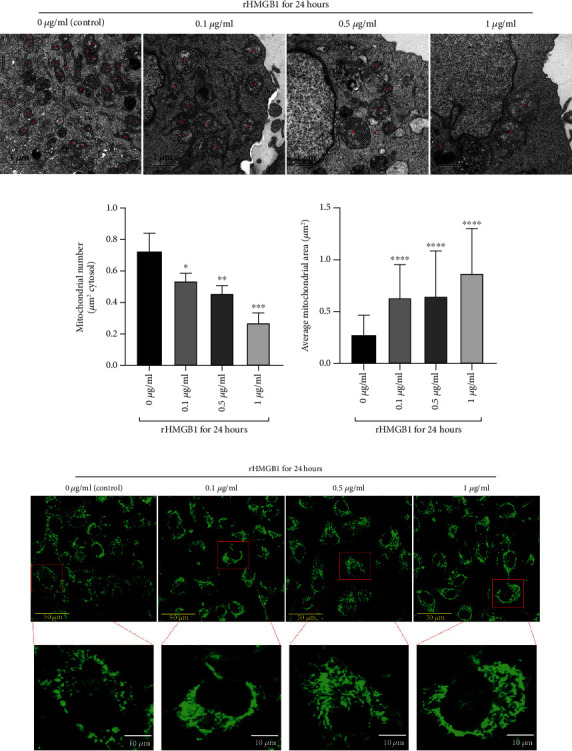
rHMGB1 incubation promoted mitochondrial fusion in EA.hy926 endothelial cells. (a) The representative TEM images of mitochondria of EA.hy926 endothelial cells treated with different concentrations (control, 0.1 *μ*g/ml, 0.5 *μ*g/ml, and 1 *μ*g/ml) of rHMGB1. The magnification is 5900. Scale bar: 1 *μ*m. (b) The mitochondrial number changes under TEM in EA.hy926 endothelial cells treated with different concentrations (control, 0.1 *μ*g/ml, 0.5 *μ*g/ml, and 1 *μ*g/ml) of rHMGB1. (c) The average mitochondrial area (*μ*m^2^) under TEM of EA.hy926 endothelial cells treated with different concentrations (control, 0.1 *μ*g/ml, 0.5 *μ*g/ml, and 1 *μ*g/ml) of rHMGB1. For comparison of average mitochondrial area, at least 30 mitochondria of 10 cells per group were calculated; for comparison of mitochondrial number, at least 10 cells per group were counted. (d) EA.hy926 endothelial cells were stained with mitochondria-specific fluorescent dye MitoTracker Green FM and imaged under a Leica SP8 confocal microscope. The magnification is 630. Scale bar (original): 50 *μ*m; scale bar (zoom): 10 *μ*m. TEM: transmission electron microscopy. ^∗^*P* < 0.05 versus control group; ^∗∗^*P* < 0.01 versus control group; ^∗∗∗^*P* < 0.001 versus control group; ^∗∗∗∗^*P* < 0.0001 versus control group.

To investigate the role of HMGB1 in mitochondrial morphology, EA.hy926 cells were incubated with rHMGB1 to investigate the number and morphology of cytosol mitochondrion by TEM and fluorescent tracing, respectively. Compared to the control group, the average numbers of mitochondrion in cytoplasm under TEM were significantly decreased in a dose-dependent manner as incubating with rHMGB1 for 24 hours (Figures 1(a) and 1(b)). In contrast, the average sizes of mitochondrion under TEM were significantly increased in cells incubated with rHMGB1 in comparison to the control group (Figures 1(a) and 1(c)). Meanwhile, profoundly tubulated mitochondria were found under a confocal microscope in rHMGB1-treated cells (Figure 1(d)). Taken together, the results suggested that rHMGB1 might trigger mitochondrial fusion in EA.hy926 cells.

### 3.2. Exogenous rHMGB1 Incubation Influenced the Expressions of Mitochondrial Dynamics-Associated Proteins

**Figure 2:**
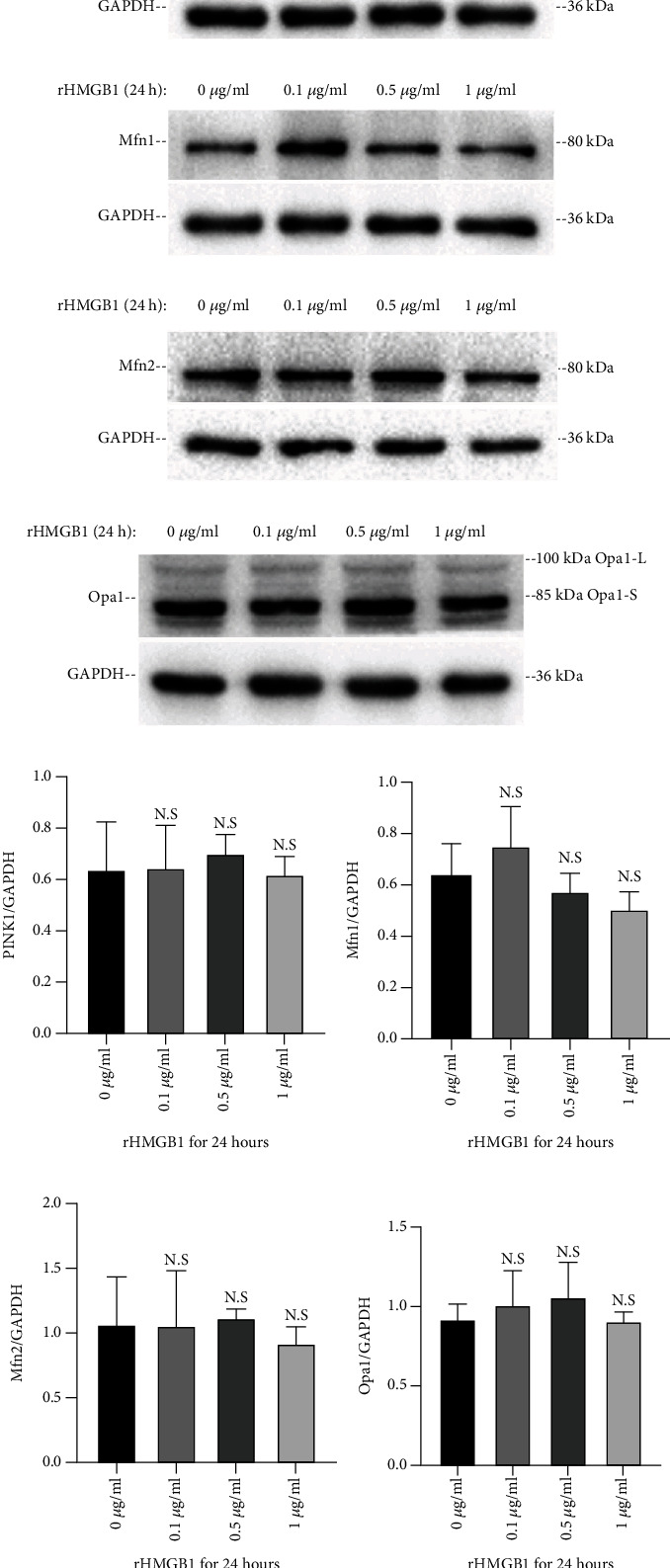
rHMGB1 had no significant effect on the protein expression of PINK1, Mfn1, Mfn2, and Opa1. (a–d) Representative immunoblotting bands of PINK1, Mfn1, Mfn2, and Opa1 and the matching internal standard GAPDH. (e–h) The densitometric analysis of relative PINK1, Mfn1, Mfn2, and Opa1 expression referenced to respective matching GAPDH. GAPDH: glyceraldehyde-3-phosphate dehydrogenase. Data were expressed as the mean ± SD.

**Figure 3:**
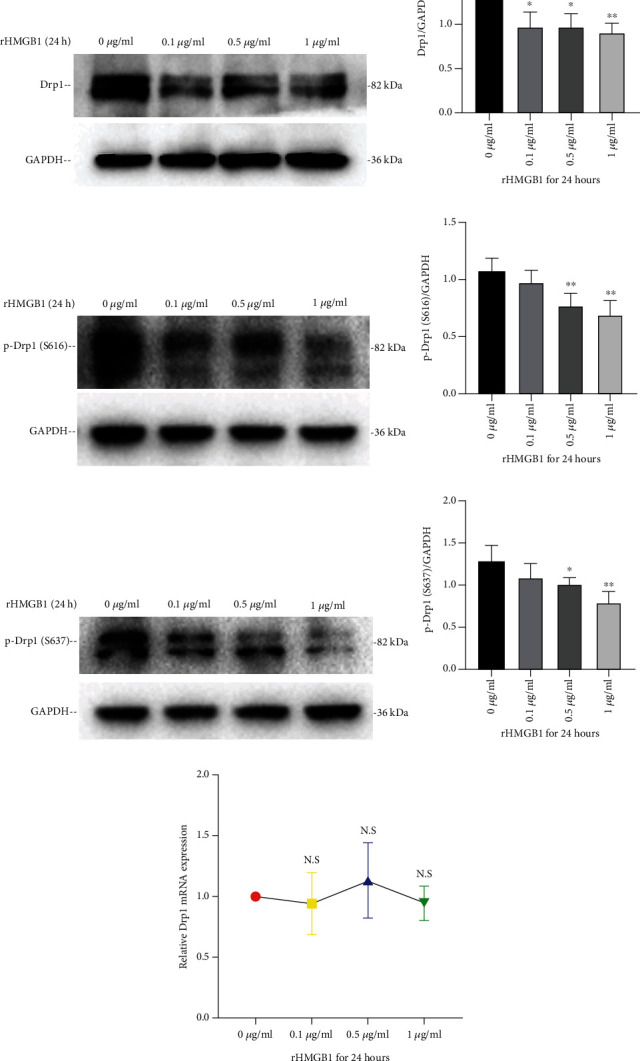
Incubation with rHMGB1 reduced the protein expression of Drp1. (a) Representative immunoblotting bands of Drp1 and the matching internal standard GAPDH. (b) The densitometric analysis of relative Drp1 expression referenced to GAPDH. (c) Representative immunoblotting bands of p-Drp1-S616 and the matching internal standard GAPDH. (d) The densitometric analysis of relative p-Drp1-S616 expression referenced to GAPDH. (e) Representative immunoblotting bands of p-Drp1-S637 and the matching internal standard GAPDH. (f) The densitometric analysis of relative p-Drp1-S637 expression referenced to GAPDH. (g) rHMGB1 had no significant effect on the expression of Drp1 mRNA expression. The amount of target mRNAs was normalized to respective internal standard GAPDH mRNA; relative fold was calculated based on the ratio of the normalized values of the cells treated with rHMGB1 to that of controls (2^-*ΔΔ*Ct^). GAPDH: glyceraldehyde-3-phosphate dehydrogenase. Data were expressed as the mean ± SD; ^∗^*P* < 0.05 versus control group and ^∗∗^*P* < 0.01 versus control group.

To clarify the potential mechanism of mitochondrial fusion induced by rHMGB1, the expressions of profusion (Mfn1, Mfn2, and Opa1), profission (Drp1), and mitophagy (PINK1)-associated proteins were determined in EA.hy926 cells, respectively. As a result, there was no significant difference of Mfn1, Mfn2, Opa1, and PINK1 protein expressions between the cells incubated with either rHMGB1 or negative control (Figures 2(a)–2(h)). However, rHMGB1 incubation significantly downregulated the Drp1 protein expression in cells with a dose-dependent manner compared to the negative control (*P* < 0.05, Figures 3(a) and 3(b)). Furthermore, the two phosphorylated subtypes, p-Drp1-S616 and p-Drp1-S637, were also explored. Consistently, compared to the negative control, rHMGB1 incubation for 24 hours at the doses of 0.5 *μ*g/ml and 1 *μ*g/ml significantly downregulated p-Drp1-S616 and p-Drp1-S637 expressions in EA.hy926 cells (*P* < 0.05, Figures 3(c)–3(f)).

Protein expression may be also determined at the gene transcriptional level. To clarify whether rHMGB1 reduces the expression of Drp1 gene at the transcription level, RT-qPCR was performed. As a result, we found that different concentrations (0.1 *μ*g/ml, 0.5 *μ*g/m, and 1 *μ*g/ml) of rHMGB1 incubation for 24 hours had no effect on the mRNA expression level of Drp1 gene in endothelial cells (Figure 3(g)), indicating that rHMGB1 downregulating the expression of Drp1 protein was not at the transcription level, but at the posttranslational level.

### 3.3. Inhibition of CXCR4 but Not TLR2, TLR4, or RAGE Abolished rHMGB1-Induced Drp1 Downregulation and Mitochondrial Fusion

**Figure 4:**
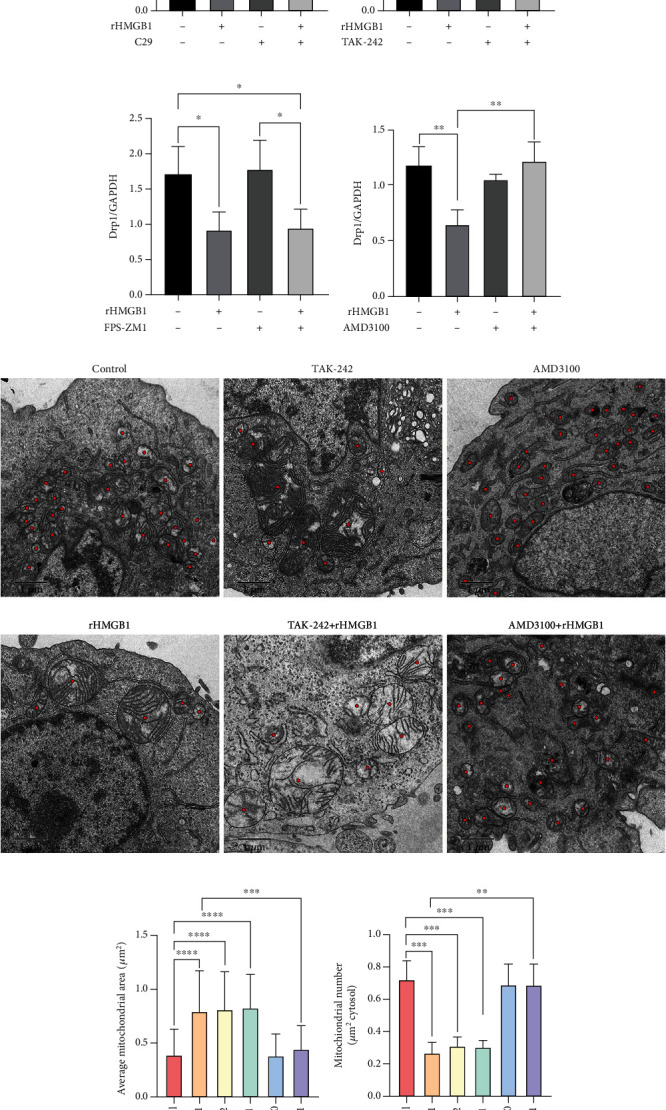
Blocking CXCR4 reversed rHMGB1-induced downregulation of Drp1 and mitochondrial fusion. (a–d) Representative immunoblotting bands of Drp1 of cells pretreated with C29, TAK-242, FPS-ZM1, and AMD3100 and the matching internal standard GAPDH. (e–h) The densitometric analysis of relative Drp1 expression of cells pretreated with C29, TAK-242, FPS-ZM1, and AMD3100 referenced to respective matching GAPDH. (i) The representative TEM images of mitochondrial morphology of cells preexposed to TAK-242, AMD3100, and subsequent rHMGB1. (j) The average mitochondrial area (*μ*m^2^) changes under TEM of cells preexposed to TAK-242, AMD3100, and subsequent rHMGB1. (k) The mitochondrial number changes under TEM in cells preexposed to TAK-242, AMD3100, and subsequent rHMGB1. (l) Mitochondrial morphology of cells preexposed to C29, TAK-242, FPS-ZM1, AMD3100, and subsequent rHMGB1. Cells were subjected to fluorescent staining with MitoTracker Green FM and observed by a Leica SP8 confocal laser scanning microscope. Scale bar: 10 *μ*m. GAPDH: glyceraldehyde-3-phosphate dehydrogenase; TEM: transmission electron microscopy. Data were expressed as the mean ± SD. For comparison of the average mitochondrial area, at least 30 mitochondria of 10 cells per group were calculated; for comparison of mitochondrial number, at least 10 cells per group were counted. ^∗^*P* < 0.05, ^∗∗^*P* < 0.01, ^∗∗∗^*P* < 0.001, and ^∗∗∗∗^*P* < 0.0001.

HMGB1 acts as a pleiotropic cytokine that plays its biological role through a variety of transmembrane receptors, including TLR2, TLR4, RAGE, and CXCR4. Specific antagonists C29 (for TLR2), TAK-242 (for TLR4), FPS-ZM1 (for RAGE), and AMD3100 (for CXCR4) were preconditioned with cells prior to exogenous rHMGB1 incubation. Cellular Drp1 expressions were significantly downregulated by rHMGB1 incubation, which were not affected by preconditioning with either C29 or FPS-ZM1 (Figures 4(a), 4(c), 4(e), and 4(g)). Interestingly, preconditioning with TAK-242 significantly reduced cellular Drp1 expression whether exogenous rHMGB1 incubation or not (Figures 4(b) and 4(f)). In contrast, preconditioning with AMD3100 significantly reversed the downregulation of Drp1 expression induced by rHMGB1 incubation (Figures 4(d) and 4(h)).

Consistently, preconditioning with C29, TAK-242, or FPS-ZM1 all could not reverse rHMGB1-triggered mitochondrial fusion in endothelial cells, but pretreatment with AMD3100 (also plerixafor octahydrochloride) abolished rHMGB1-triggered mitochondrial fusion (Figures 4(i)–4(l)). Accordingly, TAK-242 preconditioning induced mitochondrial fusion (Figures 4(i)–4(l)), which was in line with the downregulation of Drp1 expression (Figures 4(b) and 4(f)).

Generally, the activation of receptors TLR2, TLR4, or RAGE triggers cellular inflammation. To further demonstrate whether rHMGB1 has no activating effect of TLR2, TLR4, or RAGE or exerts its biological role independent of TLR2, TLR4, or RAGE, we detected the inflammatory phenotype changes of EA.hy926 cells treated with rHMGB1. Cellular NLRP3, caspase 1, and cleaved caspase 1 expressions were detected by western blot, whereas IL-1*β* concentration was determined in the culture supernatant by ELISA, respectively. Compared to the negative control, neither the expressions of NLRP3, caspase 1, and cleaved caspase 1 nor IL-1*β* concentration was altered by rHMGB1 incubation in EA.hy926 cells (Figures S2(a)-S2(c)), indicating that rHMGB1 had no significant proinflammatory effect on EA.hy926 endothelial cells, further supporting the notion that the rHMGB1 we used had no activating effect on receptor TLR2, TLR4, or RAGE.

Taken together, these results suggested that rHMGB1-induced mitochondrial fusion might be mediated by the CXCR4 pathway in EA.hy926 cells.

### 3.4. Inhibition of PSMB5 Reversed rHMGB1-Induced Decrease of Drp1 Protein Level and Mitochondrial Fusion

**Figure 5:**
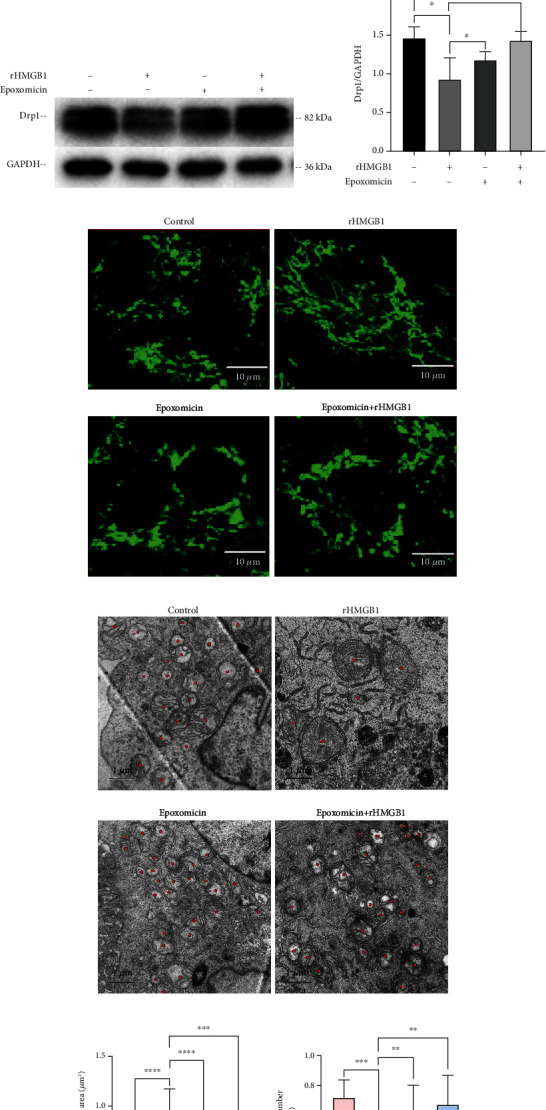
Inhibition of PSMB5 abolished rHMGB1-induced decrease in Drp1 protein and mitochondrial fusion. (a) Representative immunoblotting bands of PSMB5 of cells treated with different concentrations of rHMGB1 and the matching internal standard *β*-Tubulin. (b) The densitometric analysis of relative PSMB5 expression of cells treated with different concentrations of rHMGB1 referenced to matching *β*-Tubulin. (c) Representative immunoblotting bands of Drp1 of cells preexposed to epoxomicin (10 *μ*M) followed by rHMGB1 (1 *μ*g/ml) and the matching internal standard GAPDH. (d) The densitometric analysis of relative Drp1 expression of cells preexposed to epoxomicin followed by rHMGB1 referenced to matching GAPDH. (e) Mitochondrial morphology of cells exposed to epoxomicin (10 *μ*M) and subsequent rHMGB1 (1 *μ*g/ml). Cells were subjected to fluorescent staining with MitoTracker Green FM and observed by a Leica SP8 confocal laser scanning microscope. The magnification is 630. Scale bar: 10 *μ*m. (f) TEM images of mitochondria of EA.hy926 endothelial cells exposed to epoxomicin (10 *μ*M) and subsequent rHMGB1 (1 *μ*g/ml). The magnification is 5900. Scale bar: 1 *μ*m. (g) The average mitochondrial area (*μ*m^2^) under TEM changes of EA.hy926 endothelial cells treated with rHMGB1, epoxomicin, or both, compared with the control group. (h) The mitochondrial number changes under TEM in EA.hy926 endothelial cells treated with rHMGB1, epoxomicin, or both, compared with the control group. GAPDH: glyceraldehyde-3-phosphate dehydrogenase; TEM: transmission electron microscopy. Data were expressed as the mean ± SD. For comparison of the average mitochondrial area, at least 30 mitochondria of 10 cells per group were calculated; for comparison of mitochondrial number, at least 10 cells per group were counted. ^∗^*P* < 0.05, ^∗∗^*P* < 0.01, ^∗∗∗^*P* < 0.001, and ^∗∗∗∗^*P* < 0.0001.

Since the discrepancy of mRNA and protein expression of Drp1 was found in EA.hy926 cells incubated with rHMGB1, 20S proteasome complex subunit *β*-5 (PSMB5), one of the core components of 20S proteasome critical for Drp1 protein degradation, was further determined. As expected, exogenous rHMGB1 incubation significantly increased PSMB5 expression in EA.hy926 cells with a dose-dependent manner (Figures 5(a) and 5(b)). Furthermore, preconditioning with epoxomicin (5 *μ*M, PSMB5 specific inhibitor) for 1 hour reversed the downregulation of Drp1 expression induced by exogenous rHMGB1 (Figures 5(c) and 5(d)). Expectedly, preconditioning with epoxomicin abolished rHMGB1-induced mitochondrial fusion in EA.hy926 cells as observed by a confocal microscope (Figure 5(e)) and TEM (Figures 5(f)–5(h)). Taken together, these results indicated that rHMGB1 might trigger mitochondrial fusion in endothelial cells through PSMB5-dependent Drp1 proteolysis.

### 3.5. rHMGB1-Induced Drp1 Decrease and Mitochondrial Fusion Are NRF2 Independent

**Figure 6:**
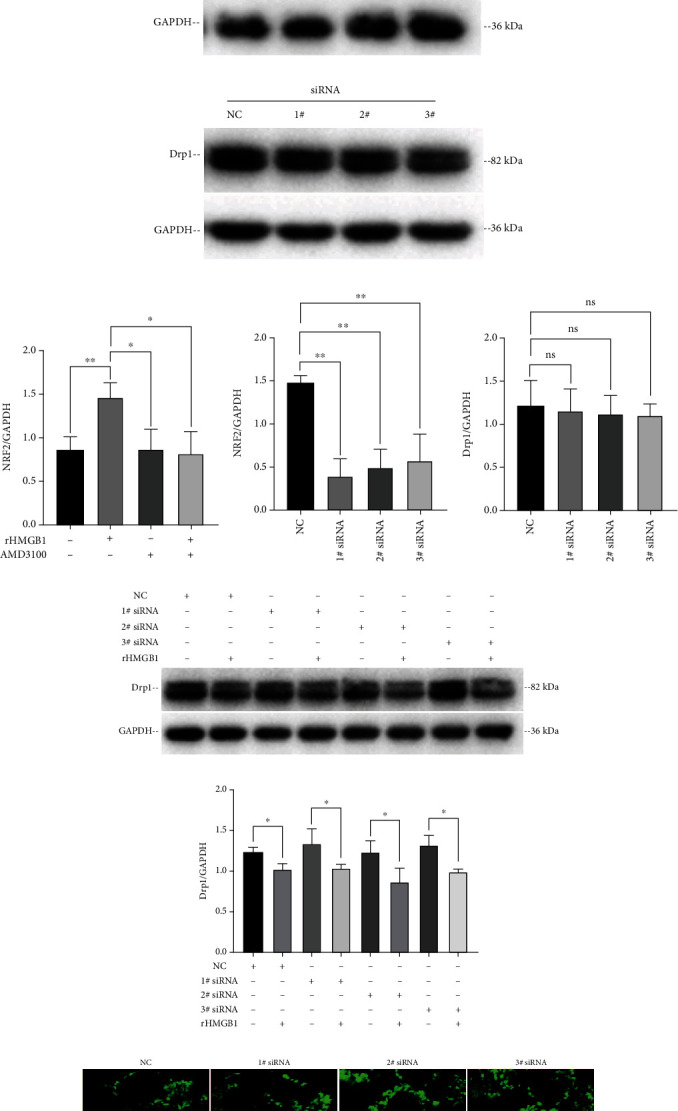
rHMGB1 induced downregulation of Drp1 and mitochondrial fusion was NRF2 independent. (a) Immunoblotting result showed that rHMGB1 upregulated the expression of NRF2, and CXCR4 antagonist AMD3100 abolished this effect. (b) Immunoblotting result demonstrated that NRF2 was successfully silenced with specific siRNAs (1# siRNA, 2# siRNA, and 3# siRNA). (c) Immunoblotting result showed that silencing the expression of NRF2 had no significant effect on the expression of Drp1. (d, e) The densitometric analysis of relative NRF2 expression of cells treated with rHMGB1, AMD3100, or siRNAs referenced to matching GAPDH. (f) The densitometric analysis of relative Drp1 expression of cells treated with siRNAs referenced to matching GAPDH. (g) Immunoblotting result showed that silencing the expression of NRF2 had no significant effect on reversing rHMGB1-induced downregulation of Drp1. (h) The densitometric analysis of relative Drp1 expression of cells treated with siRNAs and/or rHMGB1 referenced to matching GAPDH. (i) Confocal result showed that silencing the expression of NRF2 had no significant effect on mitochondrial dynamics or on reversing rHMGB1-induced mitochondrial fusion in EA.hy926 cells. Cells were subjected to fluorescent staining with MitoTracker Green FM and observed by a Leica SP8 confocal laser scanning microscope. Scale bar: 10 *μ*m. GAPDH: glyceraldehyde-3-phosphate dehydrogenase; NC: negative control. Data were expressed as the mean ± SD; ^∗^*P* < 0.05, ^∗∗^*P* < 0.01.

Nuclear factor erythroid 2-related factor 2 (NRF2) is the master antioxidant transcription factor regulating the expression of antioxidant proteins to protect against oxidative damage. It is reported that the NRF2 stress response pathway promotes mitochondrial fusion through degradation of the mitochondrial fission protein Drp1 [40]. In our study, we found that rHMGB1 treatment upregulated the expression of NRF2 through the CXCR4 signaling pathway in EA.hy926 cells (Figures 6(a) and 6(d)). This suggested that the activation of NRF2 may be involved in the rHMGB1-induced Drp1 degradation and mitochondrial fusion. We then further silenced the expression of NRF2 with specific siRNAs (Figures 6(b) and 6(e)) to define whether inhibition of NRF2 could block rHMGB1-induced Drp1 decrease and mitochondrial fusion. Unexpectedly, we found that silencing NRF2 expression had no effect on both Drp1 expression level and mitochondrial dynamics (Figures 6(c), 6(f), and 6(i)) and rHMGB1-induced decrease of Drp1 and mitochondrial fusion (Figures 6(g)–6(i)), indicating that rHMGB1-induced Drp1 decrease and mitochondrial fusion was NRF2 independent.

## 4. Discussion

HMGB1 is a multifacet protein exerting functions both inside and outside of cells, involved in a large variety of different biological processes such as inflammation, migration, invasion, proliferation, differentiation, and tissue regeneration. As a distinct proinflammatory mediator, extracellular HMGB1 may cause tissue injury and organ dysfunction in the pathogenesis of many different diseases. However, many studies have reported that HMGB1 plays an important role in tissue repair and regeneration. Meanwhile, the protective role of HMGB1 in cardiovascular pathology was also found. Limana et al. first reported that exogenous HMGB1 protein induced myocardial regeneration after infarction via enhanced cardiac C-kit+ cell proliferation and differentiation [20]. Zhou et al. recently have reported that HMGB1 protected the heart against ischemia-reperfusion injury via PI3K/Akt pathway-mediated upregulation of VEGF expression [22]. Indeed, the protection effect of HMGB1 against IR injury is not restricted in the heart, but systematically, similar protective effects were also found in IR injury of the cerebrum [41], liver [42], and kidney [43].

As aforementioned, emerging roles of HMGB1 in endothelial cells were reported; on the one hand, HMGB1 could induce endothelial dysfunction and inflammation [23, 24], inhibit endothelial cell migration [25], and enhance LDL transcytosis in endothelial cells [26]; on the other hand, HMGB1 promotes angiogenesis in endothelial cells [27] and plays a crucial role in maintaining healthy endothelial function [28].

As a protein of pleiotropic activity, HMGB1 exerts its biological activities depending on different redox forms. Structurally, HMGB1 is composed of three domains: two positively charged proximal DNA-binding domains (A box and B box) and a negatively charged carboxyl terminus. Its molecule contains three cysteine residues critical for its biological activity: two vicinal cysteines in box A (C23 and C45) and a single one in box B (C106). The fully reduced HMGB1 is characterized by all the cysteines in the thiol state and exerts chemotactic activity; the partial oxidated form leads to the formation of an intramolecular disulfide bond between the C23 and C45 and defines the disulphide-HMGB1 acting as a proinflammatory cytokine; the further oxidation of all cysteines to sulfonates characterizes the sulfonyl HMGB1 that has neither chemokine- nor cytokine-like activity [44].

Mitochondria are highly dynamic organelles that constantly undergo fission and fusion. As aforementioned, disruption of mitochondrial dynamics undermines their function and causes a variety of human diseases, including CVDs, neurodegenerative diseases, diabetes, cancer, and inflammatory diseases [34]. The role of mitochondrial dynamics in the pathogenesis of CVDs has aroused extensive attention. Changes in mitochondrial dynamics have been implicated in endothelial dysfunction, vascular smooth cell proliferation, cardiac development and differentiation, cardiomyocyte hypertrophy, myocardial IR injury, cardioprotection, and heart failure [45].

However, as an important cytokine, the effect of HMGB1 on mitochondrial dynamics remains unclear. In consideration of the important role of both HMGB1 and mitochondria dynamics in the pathogenesis of CVDs, this is undoubtedly a topic worthy of researching. As one of the major cell types of the cardiovascular system, endothelial dysfunction is involved in many CVDs, such as coronary artery disease [46], hypertension [47], aneurysm [48], and heart failure [9]. So, in this study, we targeted endothelial cells to explore the influence of HMGB1 on mitochondrial dynamics.

In our study, recombinant HMGB1 from Sigma-Aldrich (Cat# H4652) was used, which is expressed in E. coli as a N-terminal histidine-tagged protein. We found that rHMGB1 treatment induced downregulation of mitochondrial fission protein Drp1 and mitochondrial fusion, indicating as an increase of tubular mitochondria under a confocal microscope, an increase of average mitochondrial area and a decrease of the mitochondrial number under TEM. The expression levels of mitochondrial fusion proteins Mfn1, Mfn2, and Opa1 were not changed significantly, indicating that rHMGB1-induced mitochondrial fusion is caused by a decrease of Drp1. Another mitochondrial dynamics mediator PINK1 expression was also unchanged in endothelial cells treated with rHMGB1.

As for the mechanism of rHMGB1-induced downregulation of Drp1 and mitochondrial fusion, we firstly aimed at the membrane receptors for HMGB1. Extracellular HMGB1 exerts its pleiotropic biological activities by interacting with a variety of different cell surface receptors. To date, more than 10 different HMGB1 receptors have been identified and described, the most studied are focusing on RAGE, TLR2, TLR4, and CXCR4 [49, 50]. The former three receptors have a role of proinflammation [51, 52], while the latter as a chemokine receptor plays an important role in tissue regeneration and cell proliferation [53]. We found that blocking TLR2, TLR4, and RAGE receptors in EA.hy926 cells could not prevent the mitochondrial profusion effects of rHMGB1. However, pretreatment with AMD3100, the CXCR4 receptor antagonist, could completely abrogate the decrease of Drp1 protein expression and mitochondrial fusion triggered by rHMGB1, indicating that rHMGB1 promotes mitochondrial fusion through the CXCR4 receptor signaling pathway in endothelial cells. Since CXCR4 is a well-defined chemokine receptor, the rHMGB1 used in our study plays a role of chemokine. As aforementioned, fully reduced HMGB1 characterized by all the 3 cysteines in the thiol state exerts chemotactic activity; it is rational to believe that the majority of the rHMGB1 we used were in a fully reduced state. This is also supported by the fact that intracellular HMGB1 is largely in the reduced state due to the strongly negative (reducing) redox potential in cytosol and nucleus [54]. Meanwhile, no proinflammatory effect of rHMGB1 on endothelial cells was found in our study, further demonstrating that the rHMGB1 used was not in a disulphide-HMGB1 state, for the proinflammatory role of HMGB1 relying on oxidation of C23 and C45 within its molecule [55].

Our RT-PCR results showed no significant reduction of the mRNA expression level of Drp1 gene was found in EA.hy926 endothelial cells treated with rHMGB1. We speculated that rHMGB1 induced downregulation of Drp1 protein at the posttranscriptional level. PSMB5 is one of the core components of 20S proteasome, the conserved degradation machinery that is essential for maintaining cellular homeostasis [56, 57]. We found that the PSMB5 protein expression level was also upregulated in cells treated with rHMGB1, and inhibition of PSMB5 activity with epoxomicin (BU-4061T) abolished the induced downregulation of Drp1 and mitochondrial fusion, indicating that rHMGB1 downregulated the expression of Drp1 by 20S proteasome-dependent degradation.

**Figure 7:**
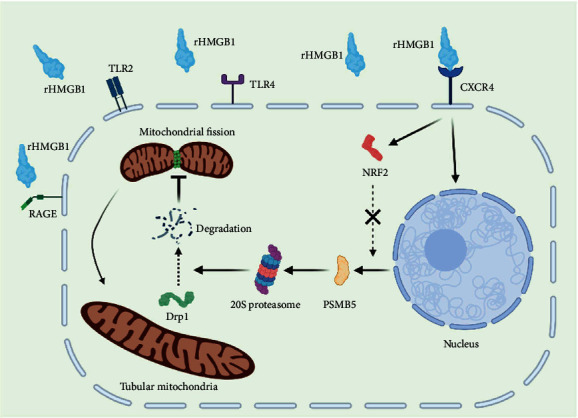
Schematic depiction of rHMGB1-induced mitochondrial fusion and the underlying mechanism. rHMGB1 combines and activates the membrane receptor CXCR4, then upregulates the expression of PSMB5 to increase the activity of 20S proteasome, leads to degradation of mitochondrial fission protein Drp1, and results in mitochondrial hyperfusion. rHMGB1: recombinant high-mobility group box 1; CXCR4: C-X-C-chemokine receptor 4; TLR2: toll-like receptor 2; TLR4: toll-like receptor 4; RAGE: receptor for advanced glycation end products; PSMB5: 20S proteasome subunit beta 5; Drp1: dynamin-related protein 1; NRF2: nuclear factor E2-related factor 2.

Several studies have reported that the Keap1-NRF2 antioxidation system plays an important role in mediating Drp1 turnover and mitochondrial dynamics. Sabouny et al. first identified that NRF2-modulated increase of proteasome activity promoted the degradation of Drp1 and mitochondrial hyperfusion [40]. Yang et al. has reported that metformin alleviated lead-induced mitochondrial fragmentation dependent on NRF2 activation [58], further supporting the role of NRF2 in promoting mitochondrial fusion. In fact, we did find that treatment with rHMGB1 upregulated the NRF2 protein level in EA.hy926 endothelial cells significantly. In consideration of the reported role of NRF2 in the turnover of Drp1, we silenced the expression of NRF2 successfully. Quite unexpectedly, silencing NRF2 had no significant effect on rHMGB1-induced reduction of Drp1 protein and mitochondrial fusion, indicating that the increase of NRF2 was not directly involved in rHMGB1-induced downregulation of Drp1 and mitochondrial fusion. Contradictory to previous reports, our results were supported by O'Mealey et al. Their study identified that sulforaphane was a NRF2-independent inhibitor of mitochondrial fission [59]. They found that sulforaphane, a potent activator of NRF2 signaling, induced a robust mitochondrial fusion in human retinal pigment epithelial (RPE-1) cells, but the expression of NRF2 was dispensable for sulforaphane-induced mitochondrial fusion. Because knockdown of NRF2 failed to counter this phenotypical change, while NRF2 stabilizing did not induce mitochondrial fusion [59]. This is in line with our results that rHMGB1-induced reduction of Drp1 protein and mitochondrial fusion was independent of NRF2 (Figure 7).

Generally, modest mitochondrial fusion is believed to be beneficial to maintaining a normal mitochondrial and cellular function, whereas mitochondrial fission is detrimental, though excessive mitochondrial fusion may be harmful. For example, many studies have indicated that heart failure and myocardial infarction are related to excessive mitochondrial fission and insufficient mitochondrial fusion [35, 60], while inhibition of Drp1 to promote mitochondrial fusion protects against myocardial ischemia-reperfusion [61, 62]. On the other hand, mitochondrial fission-fusion emerged as a key regulator of cell proliferation and differentiation. Mitochondrial fusion may promote cell proliferation, while mitochondrial fission may inhibit cell proliferation and promote cell cycle exiting to allow entry into differentiation [63, 64]. In the present study, we found that rHMGB1 caused Drp1 degradation and mitochondrial fusion through CXCR4, exerting a role of chemokine. As an important chemokine receptor, the activation of CXCR4 resulted in cell proliferation and tissue regeneration [53]. So, it is reasonable to conclude that rHMGB1-induced CXCR4-dependent mitochondrial fusion serves as a key checkpoint in its role of promoting tissue repair and regeneration. Considering the beneficial role of appropriated mitochondrial fusion, our study provided new clues for the mechanism of HMGB1-mediated cytoprotection role.

## 5. Conclusion

HMGB1 promotes mitochondrial hyperfusion through CXCR4/PSMB5-mediated Drp1 protein degradation in EA.hy926 endothelial cells in a manner of NRF2 independent, without apparent effect on the inflammatory phenotype. In the light of the important role of balanced mitochondrial dynamics in maintaining normal cellular biological function, our study sheds new light on the mechanism of HMGB1-mediated cytoprotective role.

## Data Availability

The data used to support the findings of this study are available from the corresponding author upon request.

## Supplementary Materials

Supplementary 1 Table S1: The sequences of NRF2 siRNA duplexes and negative control. Table S2: PCR primer sequences for Drp1 and GAPDH genes.

Supplementary 2 Figure S1: CD31 and vWF were strongly positive in EA.hy926 cells detected with immunofluorescent staining. (a) Cells were incubated with CD31 primary antibody, followed by Alexa Fluor 488 labeled secondary antibody, then counterstained with Hoechst. Clear green fluorescence (mostly located on cytomembrane) indicated the high expression of CD31 in EA.hy926 cells. (b) Cells were incubated with vWF primary antibody, followed by Alexa Fluor 594 labeled secondary antibody, then counterstained with Hoechst. Clear red fluorescence indicated the high expression of vWF in EA.hy926 cells. The magnification is 400. Scale bar: 50 *μ*m. CD31: cluster of differentiation 31; vWF: von Willebrand factor. Figure S2: No significant inflammatory phenotype change was found in EA.hy926 cells treated with rHMGB1. (a) Immunoblotting showed that rHMGB1 had no significant influence on the expression of NLRP3. (b) Immunoblotting showed that rHMGB1 did not increase the expression level of caspase 1 and cleaved caspase 1. (c) ELISA showed that no significant increase of IL-1*β* concentration was found in the culture supernatant of EA.hy926 cells treated with different concentrations of rHMGB1. Data were expressed as the mean ± SD. GAPDH: glyceraldehyde-3-phosphate dehydrogenase.
