# Supplementary material for: Recombinant High-Mobility Group Box 1 (rHMGB1) Promotes NRF2-Independent Mitochondrial Fusion through CXCR4/PSMB5-Mediated Drp1 Degradation in Endothelial Cells
Source: Oxid Med Cell Longev. 2021 Aug 2;2021:9993240. doi: 10.1155/2021/9993240 (PMC8358426; doi:10.1155/2021/9993240)
Supplement: Supplementary 1 — Table S1: The sequences of NRF2 siRNA duplexes and negative control. Table S2: PCR primer sequences for Drp1 and GAPDH genes. [file 9993240.f1.zip › Table S1.pdf]

Table S1 The sequences of NRF2 siRNA duplexes and negative control

siRNA	Sequence of siRNA	
	Forward	Reverse
1#	5’-GGUUGAGACUACCAUGG UUTT-3’	5’-AACCAUGGUAGUCUCAACCTT -3’
2#	5’-GACAGAAGUUGAC AAUUAUTT-3’	5’-AUAAUUGUCAACUUCUGUCTT -3’
3#	5’-CCAGAA CACUCAGUGGAAUTT-3’	5’ -AUUCCACUGAGUGUU CUGGTT -3’
Negative control	5’-UUCUCCGAACGUGUCACGUTT -3’	5’-ACGUGACACGUUCGGAGAATT -3’
