# Supplementary material for: Recombinant High-Mobility Group Box 1 (rHMGB1) Promotes NRF2-Independent Mitochondrial Fusion through CXCR4/PSMB5-Mediated Drp1 Degradation in Endothelial Cells
Source: Oxid Med Cell Longev. 2021 Aug 2;2021:9993240. doi: 10.1155/2021/9993240 (PMC8358426; doi:10.1155/2021/9993240)
Supplement: Supplementary 1 — Table S1: The sequences of NRF2 siRNA duplexes and negative control. Table S2: PCR primer sequences for Drp1 and GAPDH genes. [file 9993240.f1.zip › Table S2.pdf]

Table S2 PCR primer sequences for Drp1 and GAPDH genes

Target gene	Primer sequence	
	Sense	Antisense
Drp1	5'-GGTGGGGTTGGAGATGGTGTT-3	5'-CGCTGTTCCCGAGCAGATAGTT-3
GAPDH	5'-GTGAAGGTCGGAGTCAACGGAT-3’	5'-CCTGGAAGATGGTGATGGGATT-3’
