# Supplementary figures and images for: Recombinant High-Mobility Group Box 1 (rHMGB1) Promotes NRF2-Independent Mitochondrial Fusion through CXCR4/PSMB5-Mediated Drp1 Degradation in Endothelial Cells
Source: Oxid Med Cell Longev. 2021 Aug 2;2021:9993240. doi: 10.1155/2021/9993240 (PMC8358426; doi:10.1155/2021/9993240)

CD31

Hoechst

Merge

(a)

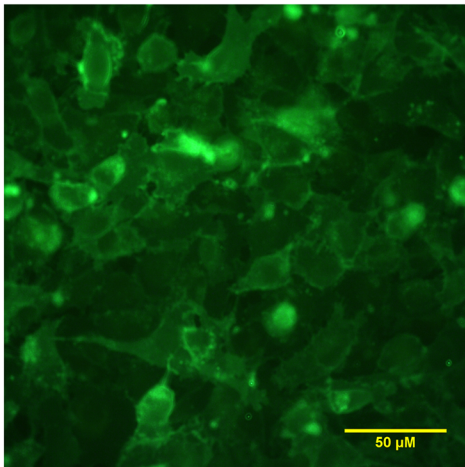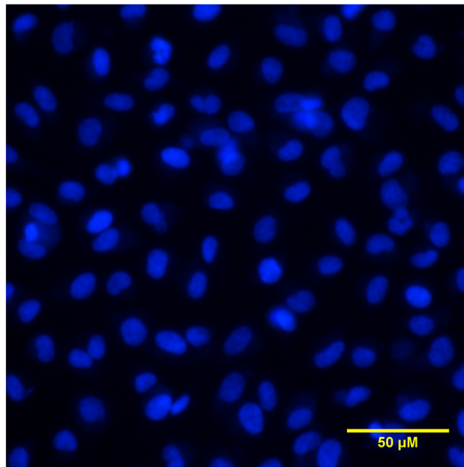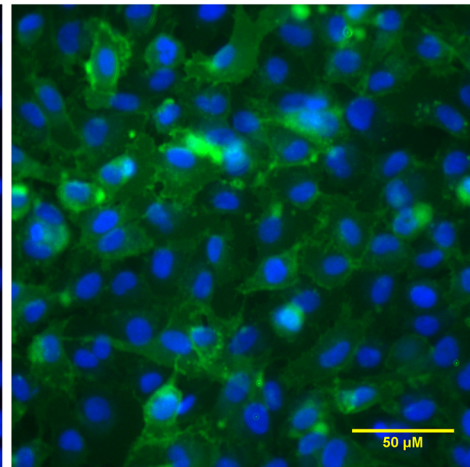

vWF

Hoechst

Merge

(b)

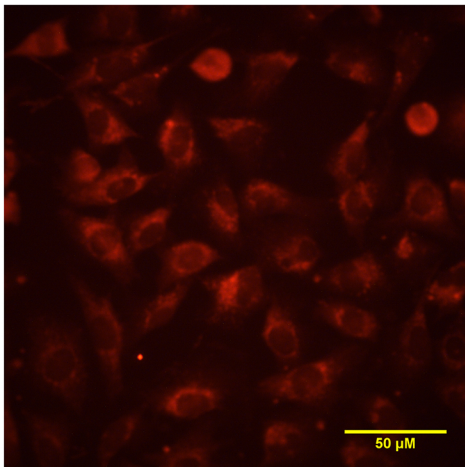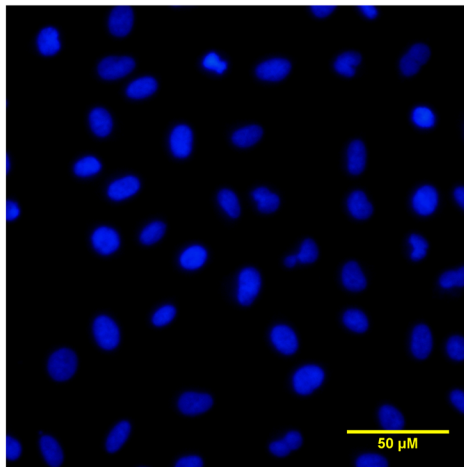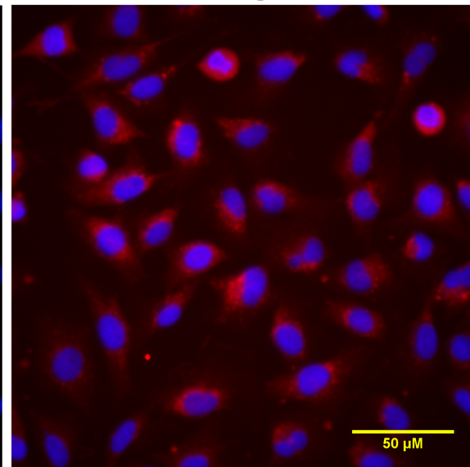

Supplement: Supplementary 2 — Figure S1: CD31 and vWF were strongly positive in EA.hy926 cells detected with immunofluorescent staining. (a) Cells were incubated with CD31 primary antibody, followed by Alexa Fluor 488 labeled secondary antibody, then counterstained with Hoechst. Clear green fluorescence (mostly located on cytomembrane) indicated the high expression of CD31 in EA.hy926 cells. (b) Cells were incubated with vWF primary antibody, followed by Alexa Fluor 594 labeled secondary antibody, then counterstained with Hoechst. Clear red fluorescence indicated the high expression of vWF in EA.hy926 cells. The magnification is 400. Scale bar: 50 μm. CD31: cluster of differentiation 31; vWF: von Willebrand factor. Figure S2: No significant inflammatory phenotype change was found in EA.hy926 cells treated with rHMGB1. (a) Immunoblotting showed that rHMGB1 had no significant influence on the expression of NLRP3. (b) Immunoblotting showed that rHMGB1 did not increase the expression level of caspase 1 and cleaved caspase 1. (c) ELISA showed that no significant increase of IL-1β concentration was found in the culture supernatant of EA.hy926 cells treated with different concentrations of rHMGB1. Data were expressed as the mean ± SD. GAPDH: glyceraldehyde-3-phosphate dehydrogenase. [file 9993240.f2.zip › figure S1.pdf]

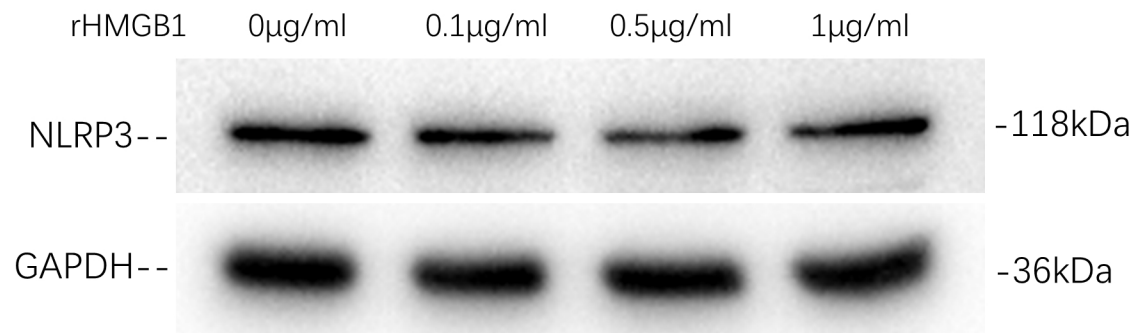

(a)

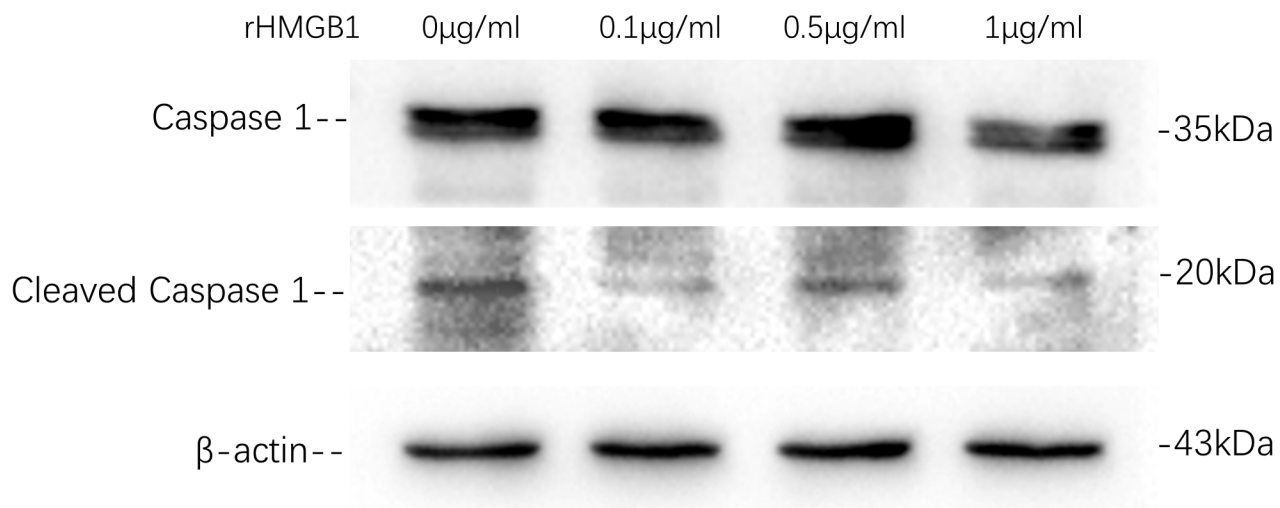

(b)

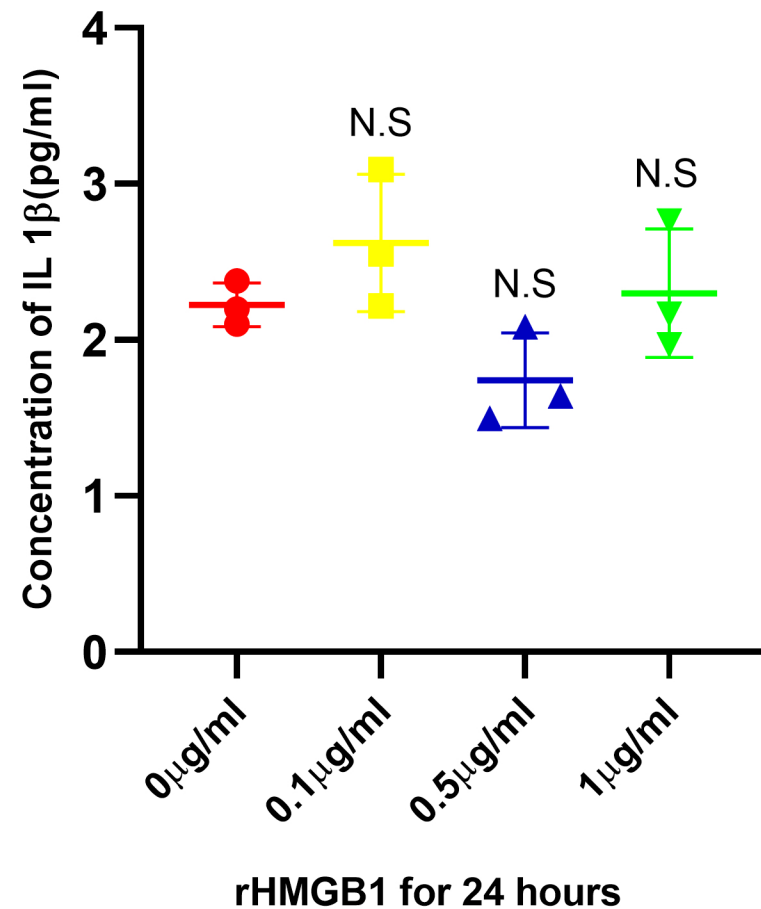

(c)

Supplement: Supplementary 2 — Figure S1: CD31 and vWF were strongly positive in EA.hy926 cells detected with immunofluorescent staining. (a) Cells were incubated with CD31 primary antibody, followed by Alexa Fluor 488 labeled secondary antibody, then counterstained with Hoechst. Clear green fluorescence (mostly located on cytomembrane) indicated the high expression of CD31 in EA.hy926 cells. (b) Cells were incubated with vWF primary antibody, followed by Alexa Fluor 594 labeled secondary antibody, then counterstained with Hoechst. Clear red fluorescence indicated the high expression of vWF in EA.hy926 cells. The magnification is 400. Scale bar: 50 μm. CD31: cluster of differentiation 31; vWF: von Willebrand factor. Figure S2: No significant inflammatory phenotype change was found in EA.hy926 cells treated with rHMGB1. (a) Immunoblotting showed that rHMGB1 had no significant influence on the expression of NLRP3. (b) Immunoblotting showed that rHMGB1 did not increase the expression level of caspase 1 and cleaved caspase 1. (c) ELISA showed that no significant increase of IL-1β concentration was found in the culture supernatant of EA.hy926 cells treated with different concentrations of rHMGB1. Data were expressed as the mean ± SD. GAPDH: glyceraldehyde-3-phosphate dehydrogenase. [file 9993240.f2.zip › figure S2.pdf]
